# Supplementary material for: Ecological mechanisms and current systems shape the modular structure of the global oceans’ prokaryotic seascape
Source: Nat Commun. 2023 Oct 2;14:6141. doi: 10.1038/s41467-023-41909-z (PMC10545751; doi:10.1038/s41467-023-41909-z)
Supplement: Supplementary file 1 — Supplementary Information [file 41467_2023_41909_MOESM1_ESM.pdf]

Supplementary Information:

Ecological mechanisms and current systems shape the modular structure of the global oceans' prokaryotic seascape

Felix Milke[1*], Jens Meyerjürgens[1], Meinhard Simon[1,2 *]

[1] Institute for Chemistry and Biology of the Marine Environment,
University of Oldenburg, Carl von Ossietzky Str. 9-11, D-26129 Oldenburg, Germany

[2] Helmholtz Institute for Functional Marine Biodiversity at the University of Oldenburg (HIFMB),
Ammerländer Heerstraße 231, D-26129 Oldenburg, Germany

* Corresponding authors (felix.milke@uni-oldenburg.de, m.simon@icbm.de)

Supplementary Fig. 1 Shared and distinct ASVs of the 0.2-3, 3-8 and >8 µm size fraction of the prokaryotic communities in the Atlantic and Pacific Ocean.
Supplementary Fig. 2 Unique ASVs in the Atlantic and Pacific Ocean.
Supplementary Fig. 3 Richness and Effective Number of Species (inverse Simpson) of ASVs in the Atlantic and Pacific Ocean along latitudinal transects.
Supplementary Fig. 4 Relative proportions of homogeneous and heterogeneous selection.
Supplementary Fig. 5 Relative importance of ecological mechanisms for prokaryotic community assembly with increasing difference of water temperature in the Pacific and Atlantic Ocean.
Supplementary Fig. 6 Proportions of homogeneous and heterogeneous selection, homogenizing dispersal and dispersal limitation and drift on assembly of the 0.2-3, 3-8 and >8 µm prokaryotic communities for a downsampled dataset.
Supplementary Fig. 7 Proportions of homogeneous and heterogeneous selection, homogenizing dispersal and dispersal limitation and drift on assembly of the 0.2-3, 3-8 and >8 µm prokaryotic communities for the complete dataset.
Supplementary Fig. 8 Environmental preferences of prokaryotic modules along latitudinal transects in the Atlantic and Pacific Ocean.
Supplementary Fig. 9 Taxonomic composition of modules.
Supplementary Fig. 10 Constrained correspondence analysis (CCA) of module abundance in all samples from the Pacific and Atlantic Ocean transects
Supplementary Fig. 11 Constrained correspondence analysis (CCA) of ASV abundance in all samples from the Pacific and Atlantic Ocean transects.
Supplementary Fig. 12 Clustering coefficient and modularity of subnetworks of the 0.2-3, 3-8 and >8 µm size fractions of the prokaryotic communities in the upper (20 m to DCM) and lower epipelagic (DCM-200 m) of the Atlantic and Pacific Ocean.
Supplementary Fig. 13 Number of simultaneously occurring modules displayed against ASV richness per sample for upper (20 m – DCM) and lower epipelagic (DCM – 200 m) and both ocean basins.
Supplementary Fig. 14 Tracer concentrations in the global oceans as a result of a five year simulation of drifter movement.
Supplementary Fig. 15 Global analysis of absolute mean velocity in cm/s derived from the Global Drifter Program dataset including 24,971 Surface Velocity Program (SVP) drifters.
Supplementary Fig. 16 Overlap of tracer accumulation, sample modularity and effective module diversity along the Atlantic and Pacific Ocean transect for the upper epipelagic depth layer (20 m – DCM) and two different simulation times.

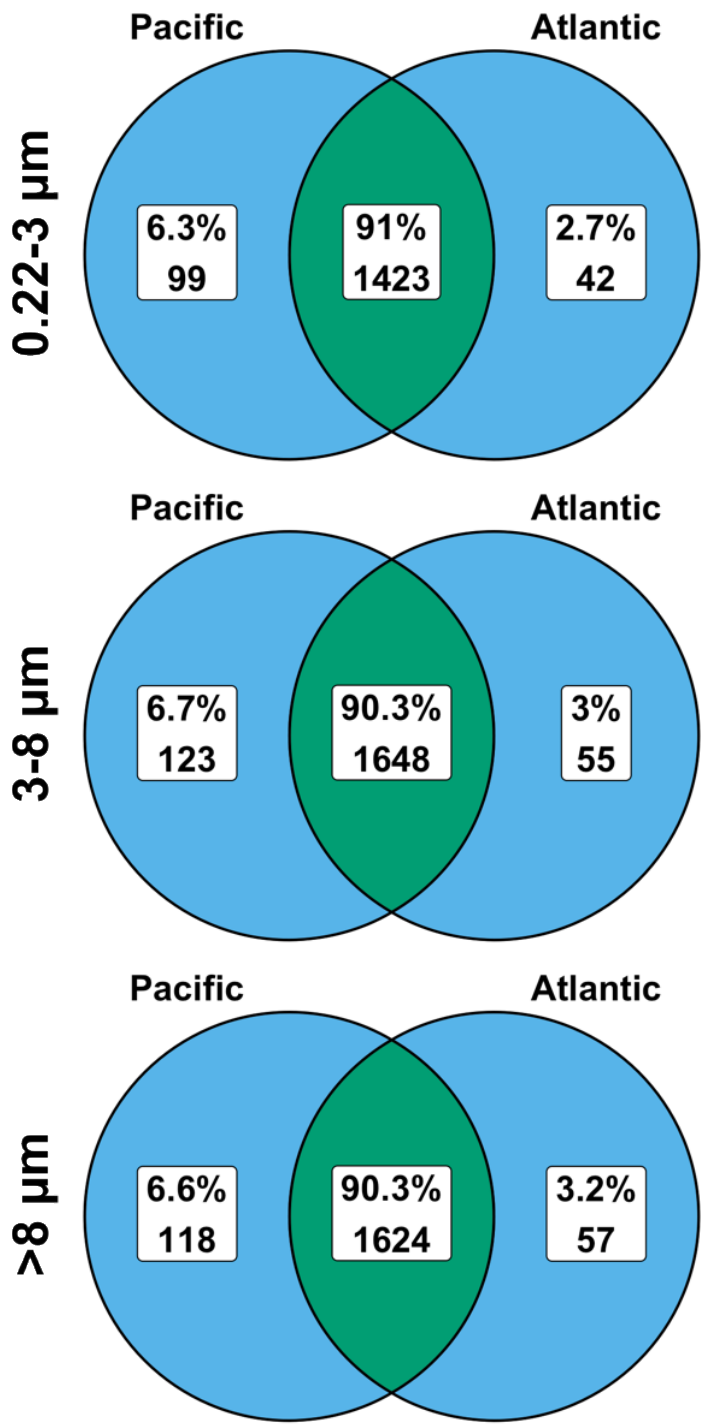

Supplementary Fig. 1 Shared and distinct ASVs of the 0.2-3, 3-8 and >8 µm size fraction of the prokaryotic communities in the Atlantic and Pacific Ocean Percentages and absolute numbers of the ASVs in the respective sections of the Venn diagrams are given.

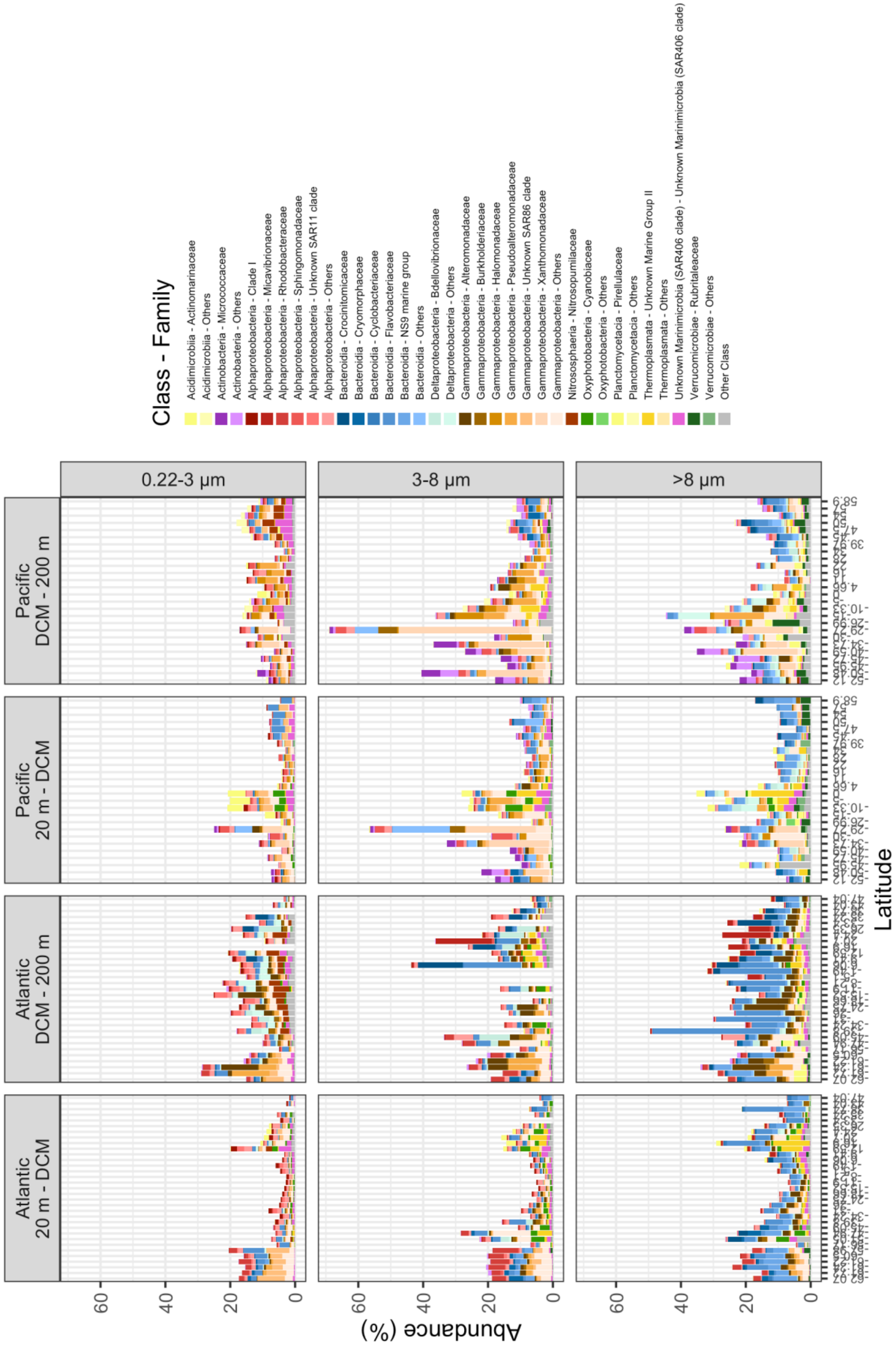

Supplementary Fig. 2 Unique ASVs in the Atlantic and Pacific Ocean Unique ASVs in the 0.2-3, 3-8 and >8 µm size fractions in the upper (20 m-DCM) and lower epipelagic (DCM-200 m) between 62°S and 47°N in the Atlantic and between 52°S and 59°N in the Pacific Ocean. Source data are provided as a Source Data file.

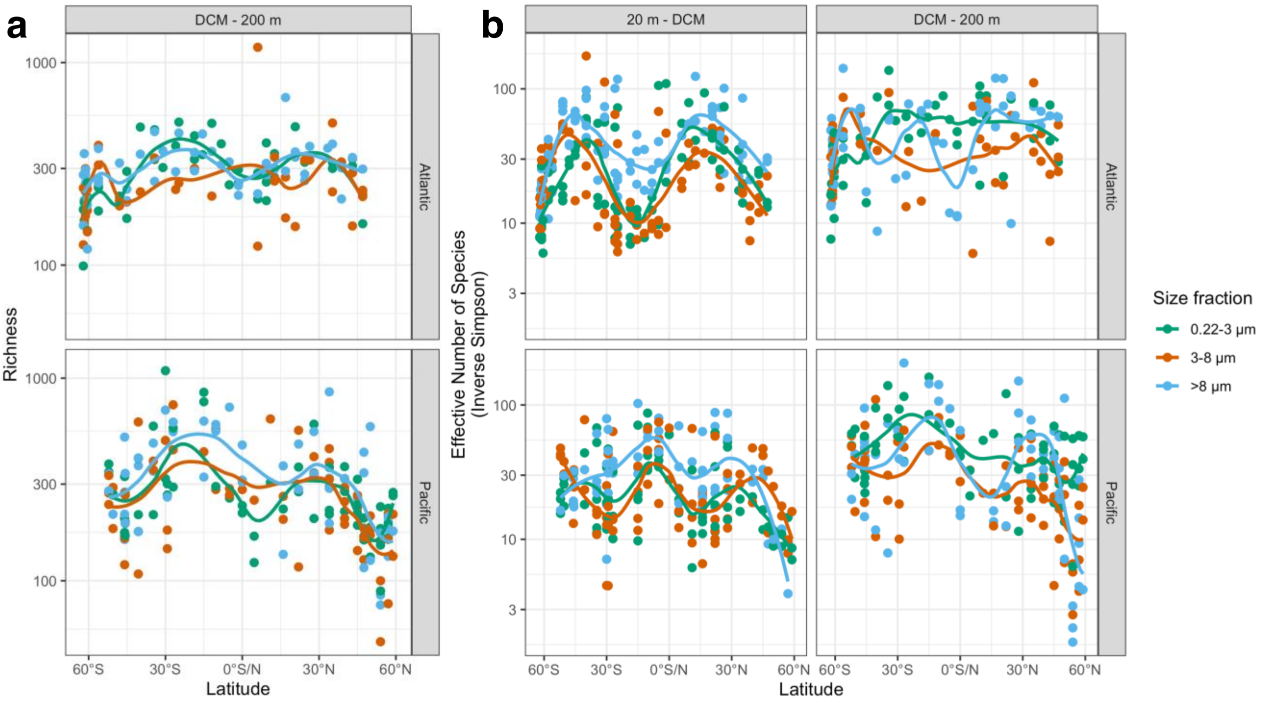

Supplementary Fig. 3 Richness and Effective Number of Species (inverse Simpson) of ASVs in the Atlantic and Pacific Ocean along latitudinal transects a Richness of ASVs of the 0.2-3, 3-8 and >8 µm size fractions of the prokaryotic communities in the lower epipelagic (DCM-200 m) of the Atlantic and Pacific Ocean. **b** As in panel a but shown are the Effective Number of Species. Points indicate single samples and the line the smoothed loess-fit. Source data are provided as a Source Data file.

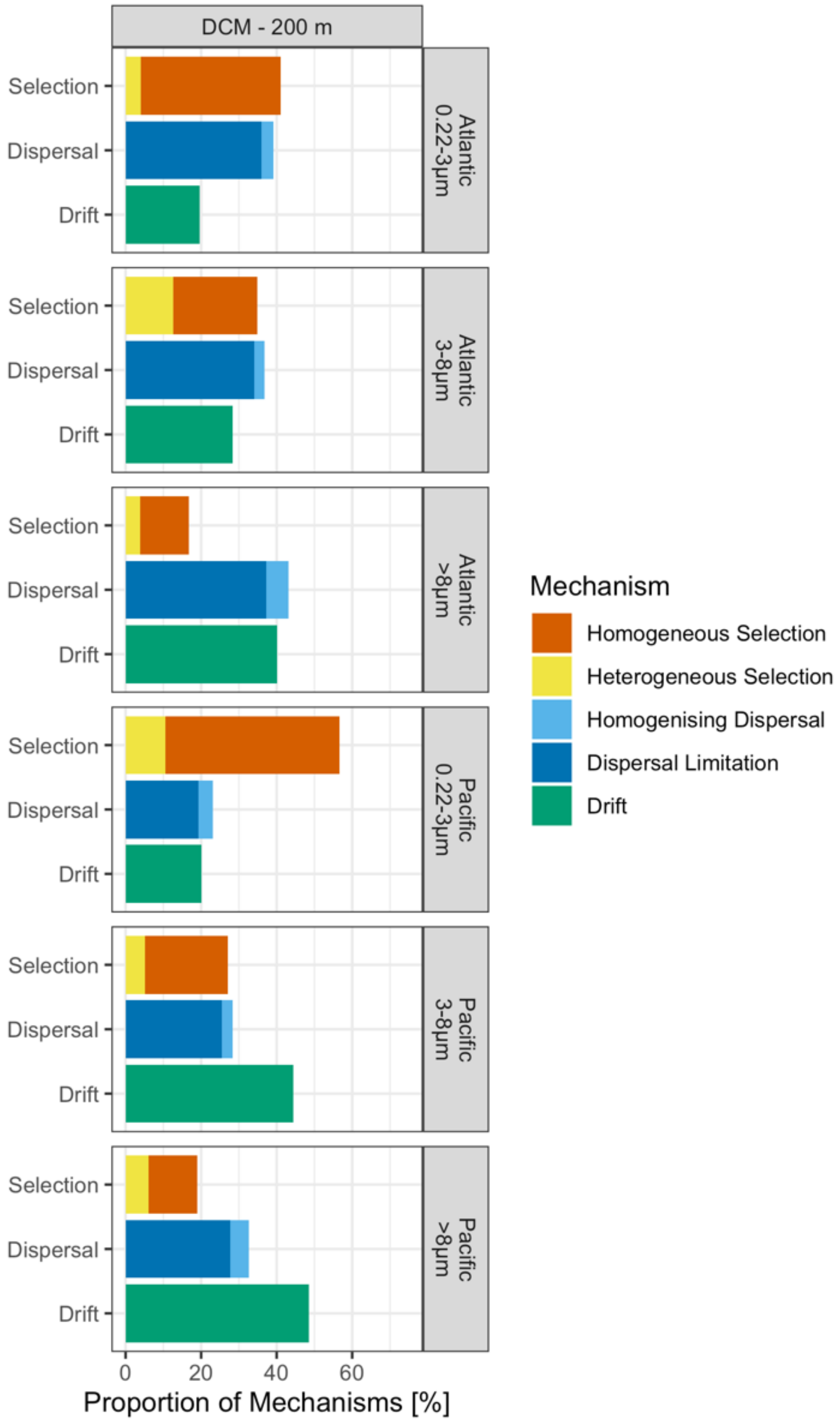

Supplementary Fig. 4 Relative proportions of homogeneous and heterogeneous selection, homogenizing dispersal and dispersal limitation and drift on community assembly of the 0.2-3, 3-8 and >8 µm prokaryotic communities in the lower epipelagic (DCM-200 m) of the Atlantic and Pacific Ocean Source data are provided as a Source Data file.

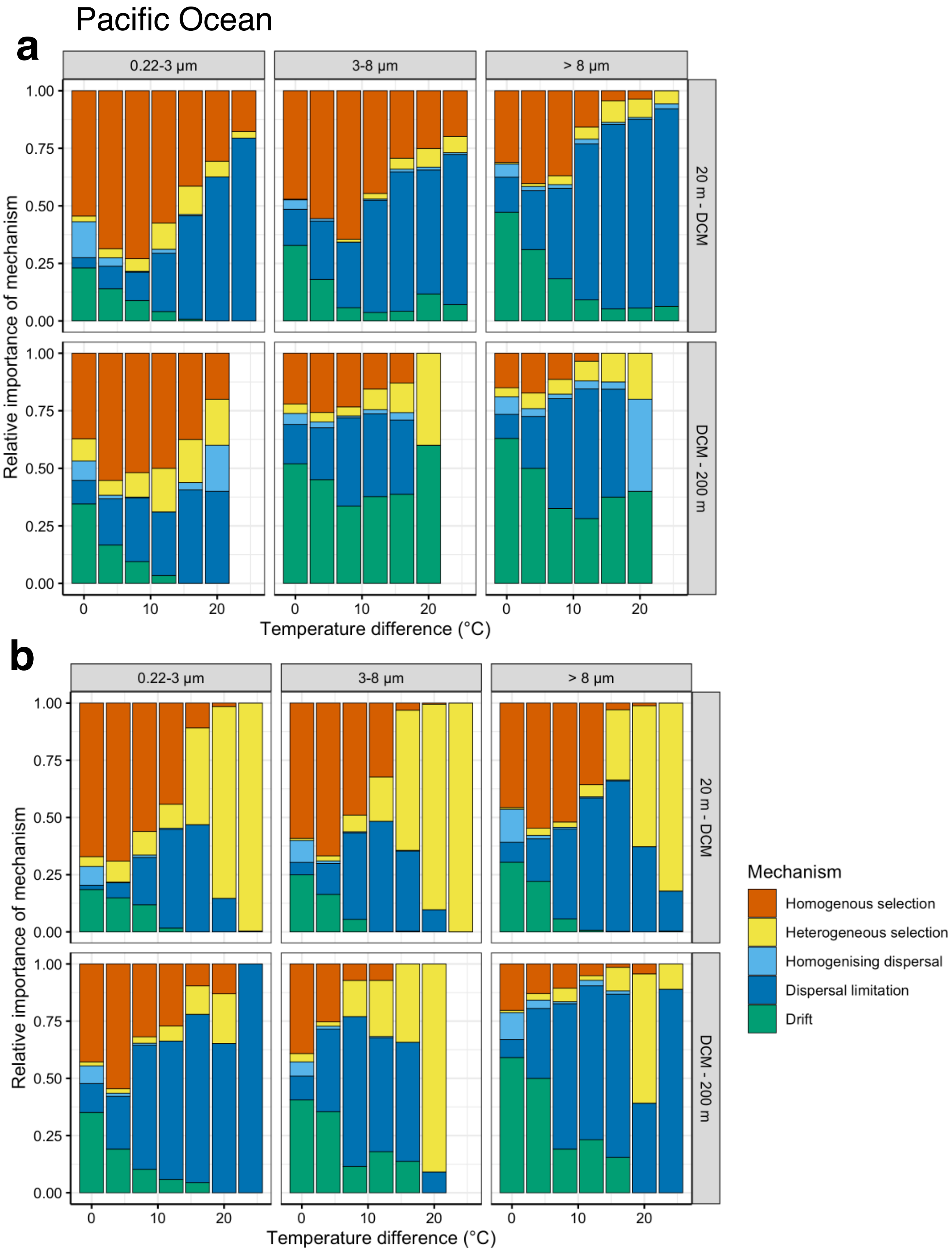

Supplementary Fig. 5 Relative importance of ecological mechanisms for prokaryotic community assembly with increasing difference of water temperature in the Pacific and Atlantic Ocean a Stacked percentages of selection, dispersal and drift of the 0.2-3, 3-8 and >8 µm size fractions for assembly of prokaryotic communities with increasing temperature differences from 0 to 28°C in the in the upper (20 m-DCM) and lower epipelagic (DCM-200 m) in Pacific Ocean. Temperature ranges of 4°C were pooled and shown in one bar (0-4, 4-8, 8-12, 12-16, 16-20, 20-24, 24-28°C). **b** Same as panel a but for the Atlantic Ocean. Source data are provided as a Source Data file.

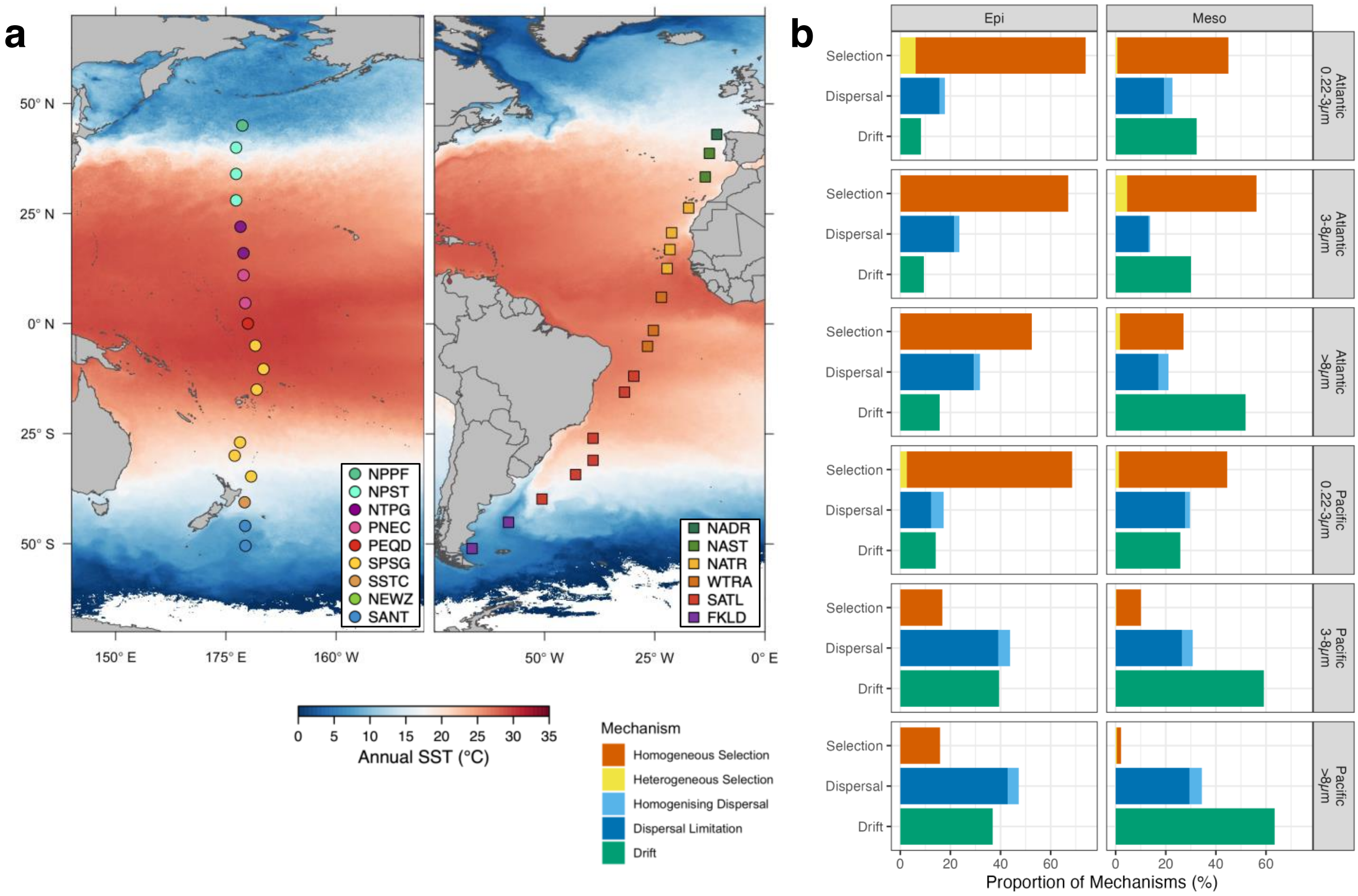

Supplementary Fig. 6 Proportions of homogeneous and heterogeneous selection, homogenizing dispersal and dispersal limitation and drift on assembly of the 0.2-3, 3-8 and >8 µm prokaryotic communities for a downsampled dataset a Maps showing station subset in the Pacific Ocean and Atlantic Ocean basins. Background colours indicate sea surface temperature and its colour code is represented below. Stations are colour coded by Longhurstian provinces as indicated in the respective legends. **b** Relative importance of the ecological mechanisms for prokaryotic community assembly separated into two depth layers (upper epipelagic: 20m – DCM and lower epipelagic: DCM – 200m) and three size-fractions. Source data are provided as a Source Data file.). Maps are modifications of previously versions published according to CC BY[1, 2].

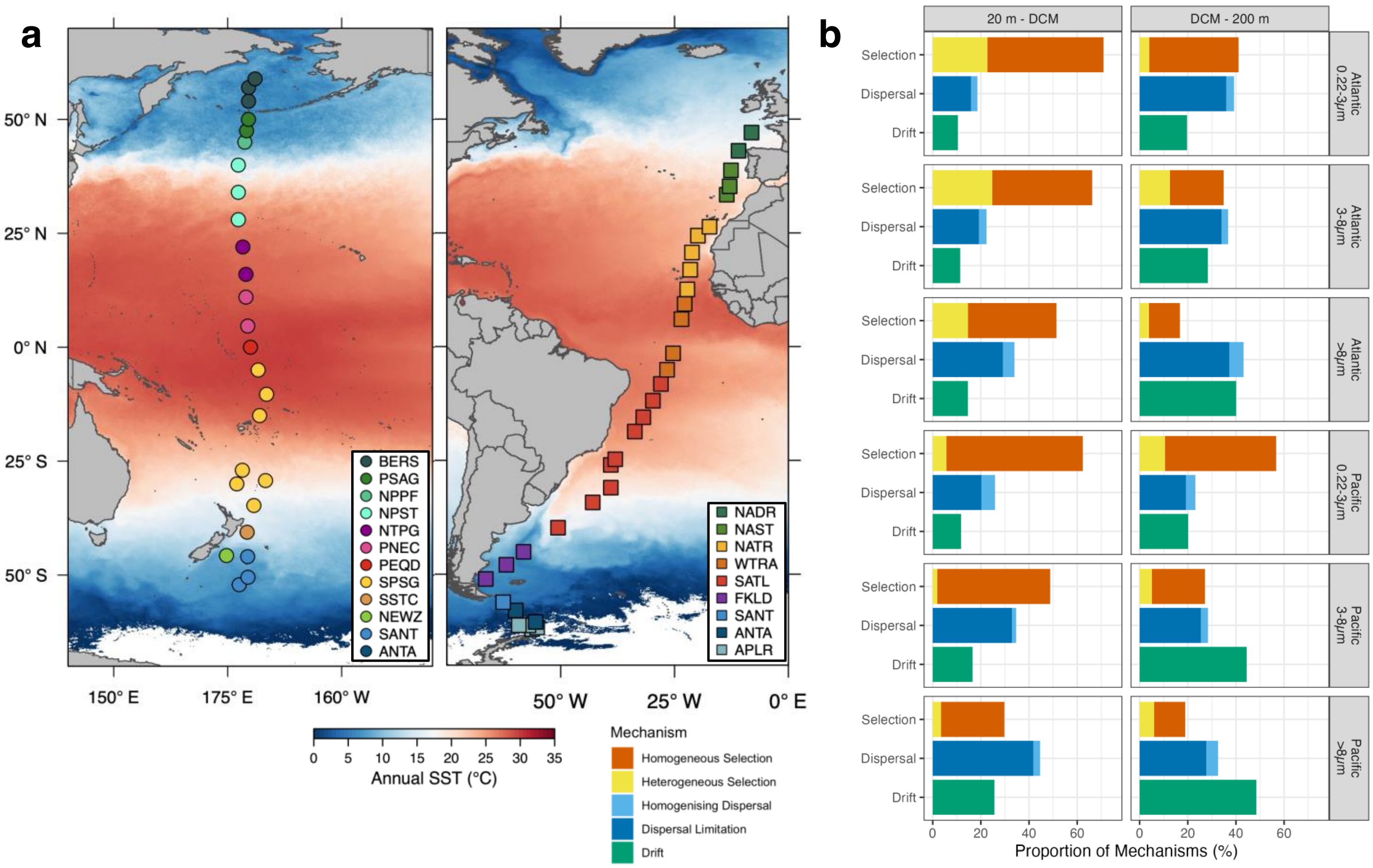

Supplementary Fig. 7 Proportions of homogeneous and heterogeneous selection, homogenizing dispersal and dispersal limitation and drift on assembly of the 0.2-3, 3-8 and >8 µm prokaryotic communities for the complete dataset **a** Maps showing station subset in the Pacific Ocean and Atlantic Ocean basins. Background colours indicate sea surface temperature and its colour code is represented below. Stations are colour coded by Longhurstian provinces as indicated in the respective legends. **b** Relative importance of the ecological mechanisms for prokaryotic community assembly separated into two depth layers (upper epipelagic: 20m – DCM and lower epipelagic: DCM – 200m) and three size-fractions. Source data are provided as a Source Data file. Maps are modifications of previously versions published according to CC BY[1, 2].

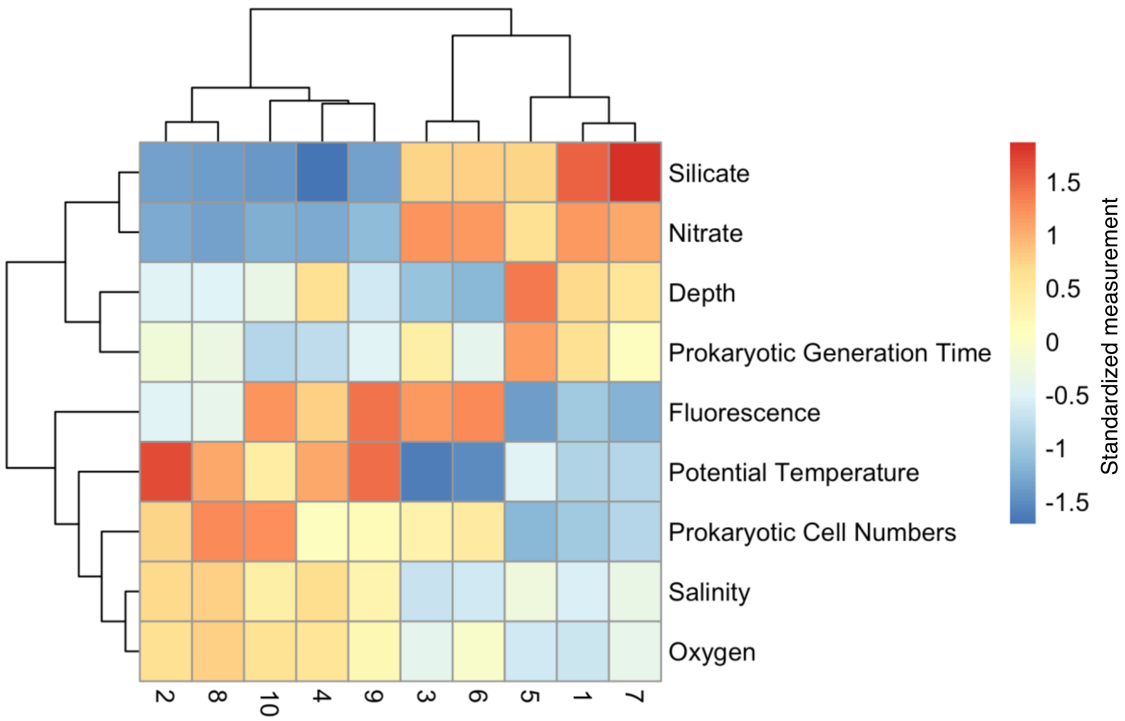

Supplementary Fig. 8 Environmental preferences of prokaryotic modules along latitudinal transects in the Atlantic and Pacific Ocean Environmental parameters are displayed in rows and co-occurrence modules in columns. Parameter values are normalized by total sum and standardized by z-scores. Rows and columns are ordered by hierarchical clustering. Color code is depicted in the color bar. Source data are provided as a Source Data file.

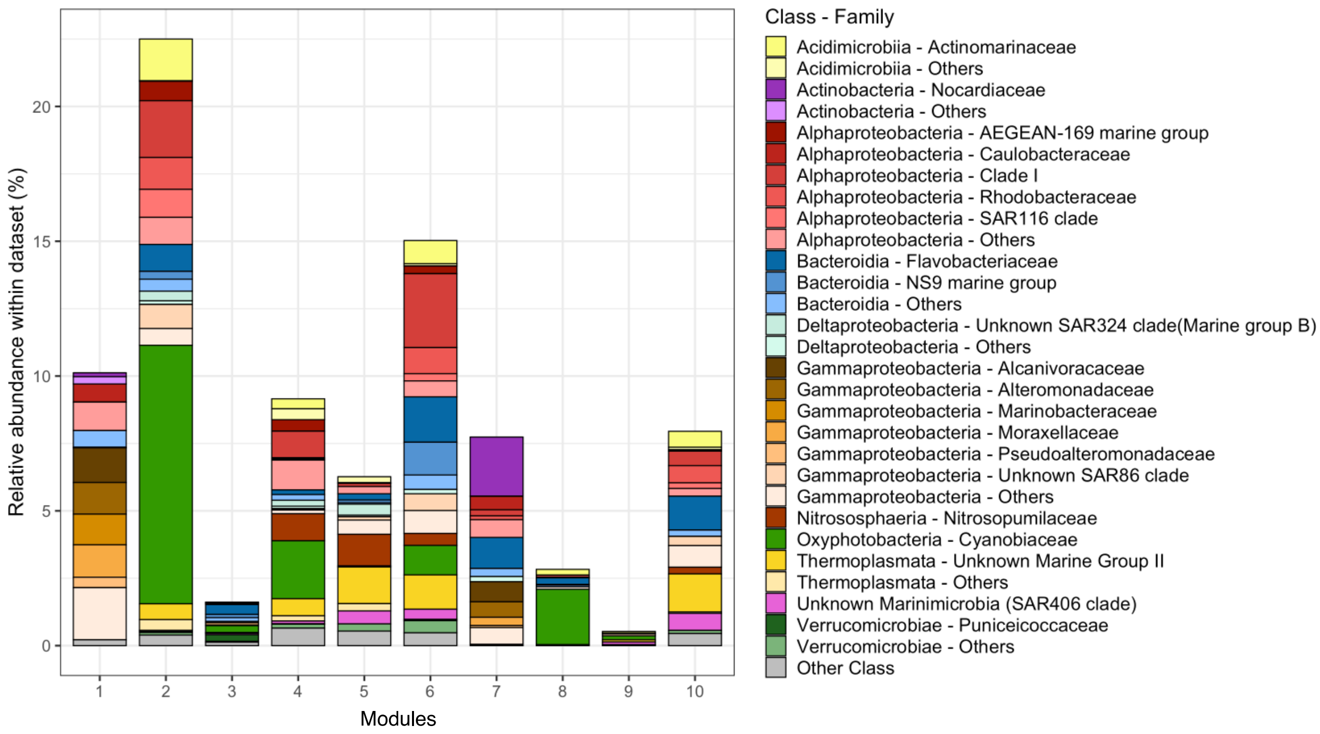

Supplementary Fig. 9 Taxonomic composition of modules Taxonomic composition of modules 1 to 10 on the class and family level. Abundance is shown as the average abundance of ASVs within the dataset of the two latitudinal transects. For a complete list of all ASVs per module see supplementary data S1.

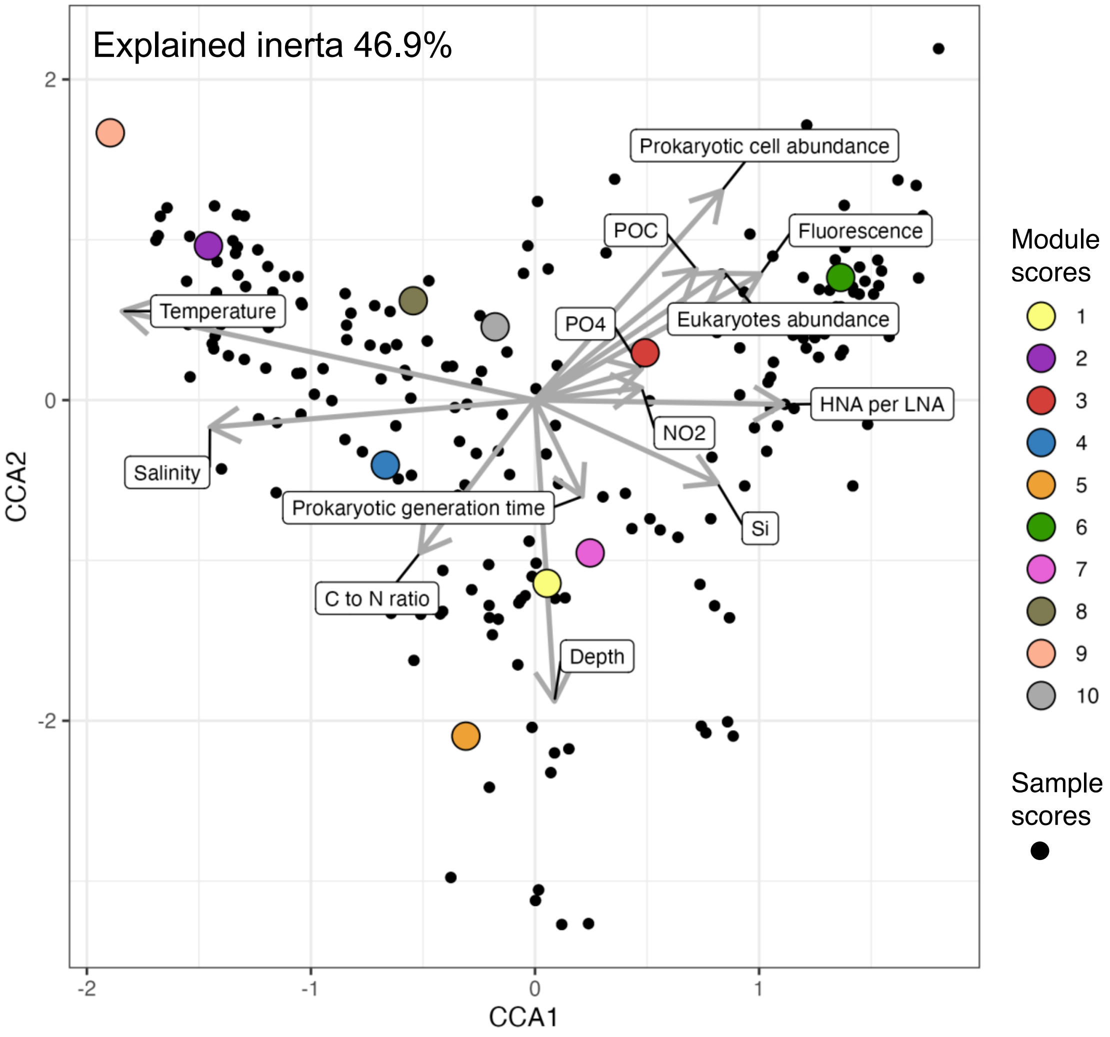

Supplementary Fig. 10 Constrained correspondence analysis (CCA) of module abundance in all samples from the Pacific and Atlantic Ocean transects Black points indicate station scores and colored points represent species scores of modules. Environmental parameters that were found to be significantly influencing module variation in the dataset were detected using stepwise forward selection of a set of abiotic and biotic environmental parameters. Explained inertia shows percentage of variation explained by underlying model.

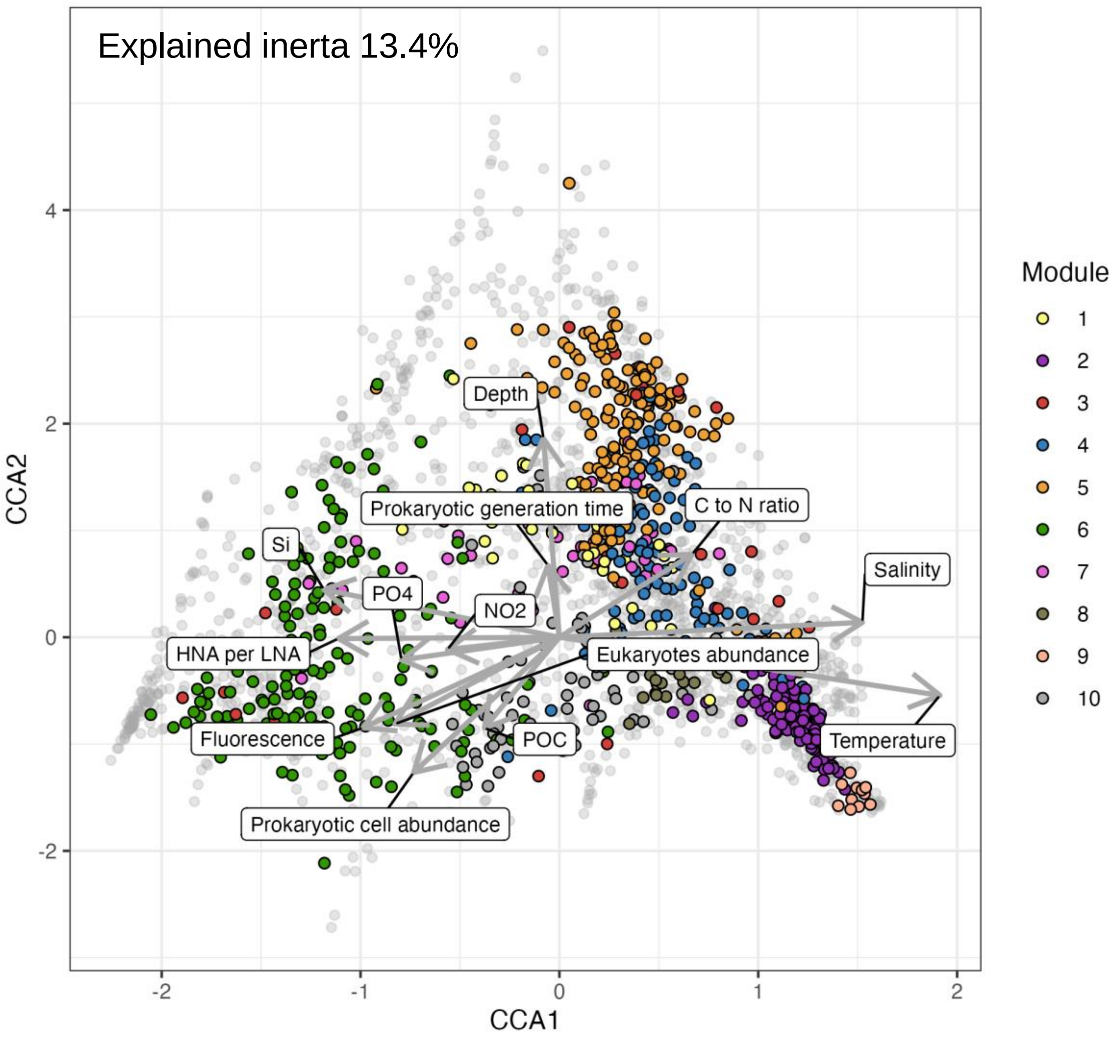

Supplementary Fig. 11 Constrained correspondence analysis (CCA) of ASV abundance in all samples from the Pacific and Atlantic Ocean transects Points indicate ASV scores and colored points represent module identity of ASVs if available. Environmental parameters that were found to be significantly influencing module variation in the dataset were detected using stepwise forward selection of a set of abiotic and biotic environmental parameters. Explained inertia shows percentage of variation explained by underlying model.

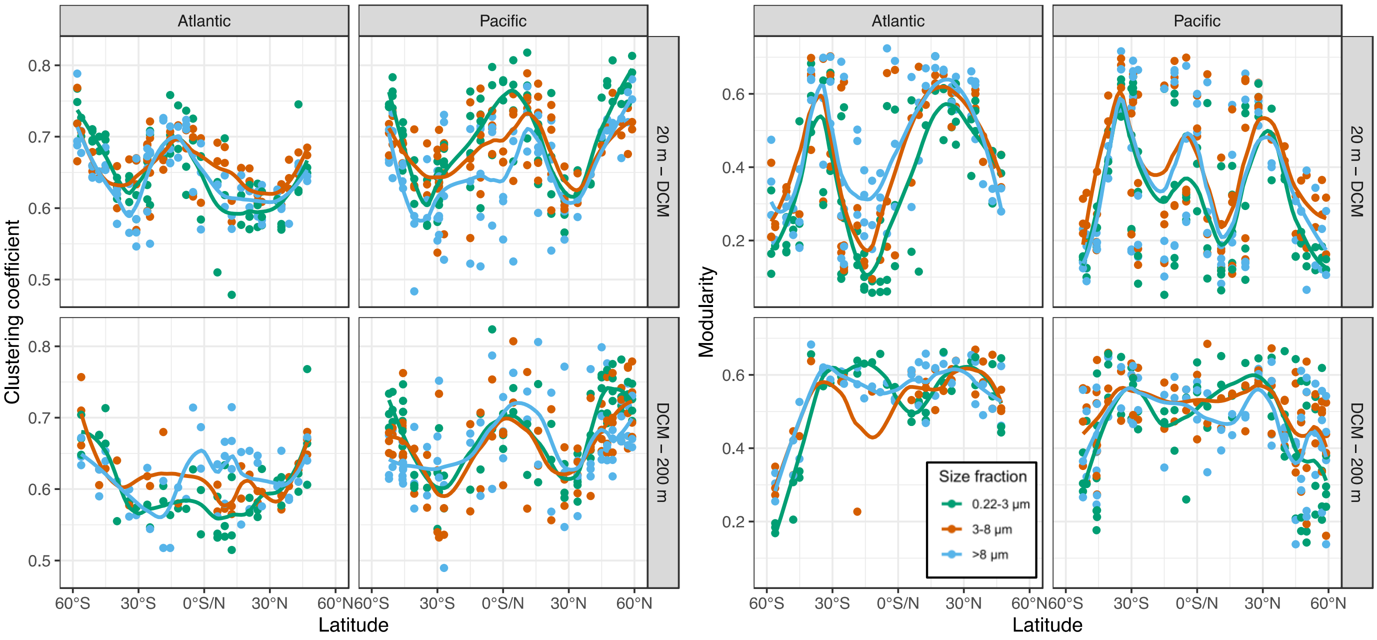

Supplementary Fig. 12 Clustering coefficient and modularity of subnetworks of the 0.2-3, 3-8 and >8 µm size fractions of the prokaryotic communities in the upper (20 m to DCM) and lower epipelagic (DCM-200 m) of the Atlantic and Pacific Ocean Points indicate single samples and the line the smoothed loess-fit. Source data are provided as a Source Data file.

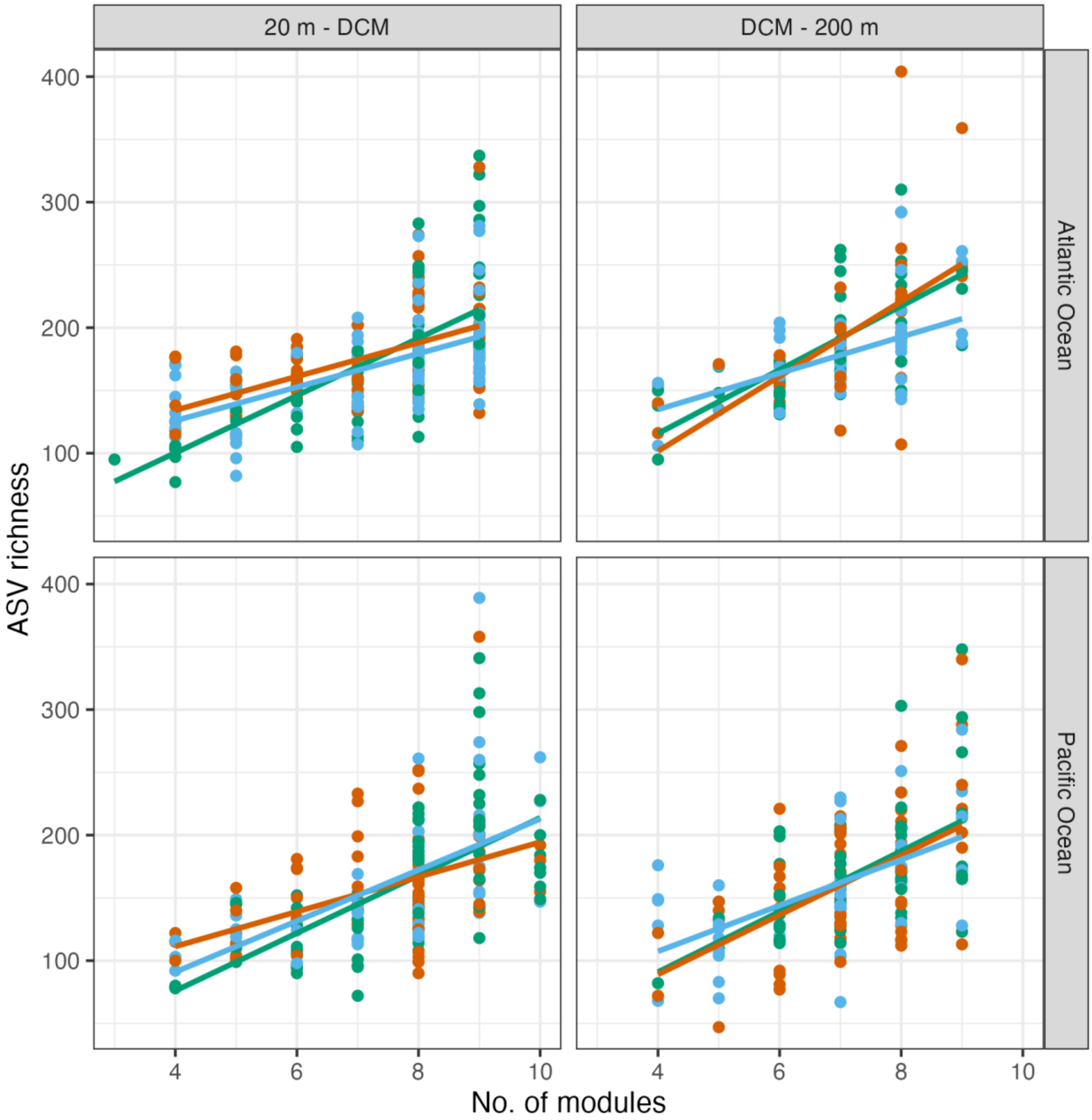

Supplementary Fig. 13 Number of simultaneously occurring modules displayed against ASV richness per sample for upper (20 m – DCM) and lower epipelagic (DCM – 200 m) and both ocean basins
Colours indicate different size-fractions and the lines represent linear fits. All linear models were highly significant (adj. p-value < 10^{-4}) and yielded good fits (0.45 < Pearson *r* < 0.692). Source data are provided as a Source Data file.

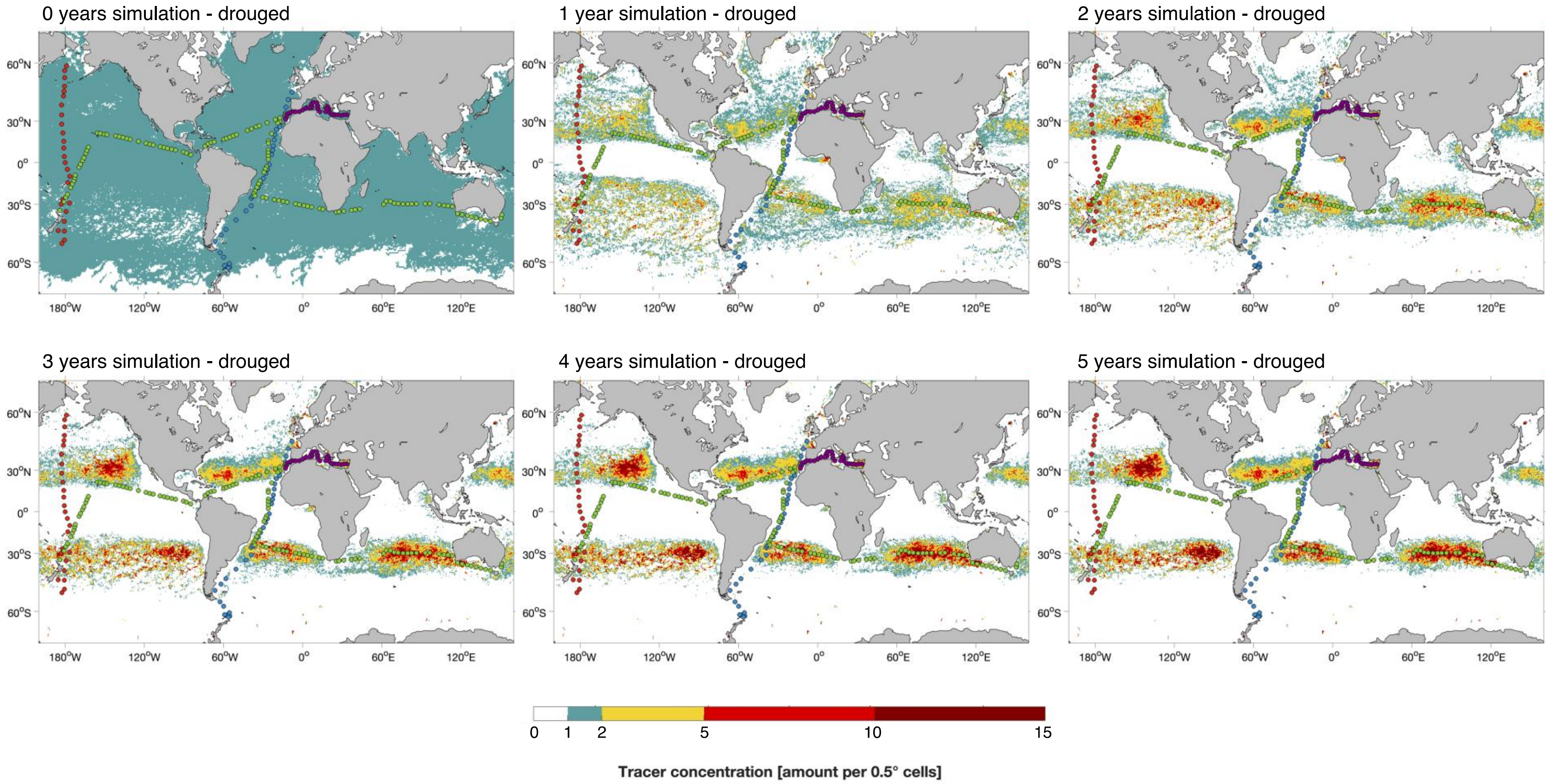

Supplementary Fig. 14 Tracer concentrations in the global oceans as a result of a five year simulation of drifter movement. **0** Initial tracer concentration. **1** Tracer concentration of the 1 year simulation. **2** Tracer concentration of the 2 year simulation. **3** Tracer concentration of the 3 year simulation. **4** Tracer concentration of the 4 year simulation. 5 Tracer concentration of the 5 year simulation. Points indicate stations of all cruises considered in this study; blue: Atlantic Ocean (RV Polarstern cruises ANTXXVIII/4 and /5), red: Pacific Ocean (RV Sonne cruises SO248 and SO254); green: Malaspina expedition; violet: Mediterranean Sea (RV Sarmiento de Gamboa). Drifter data were taken from reference 3.

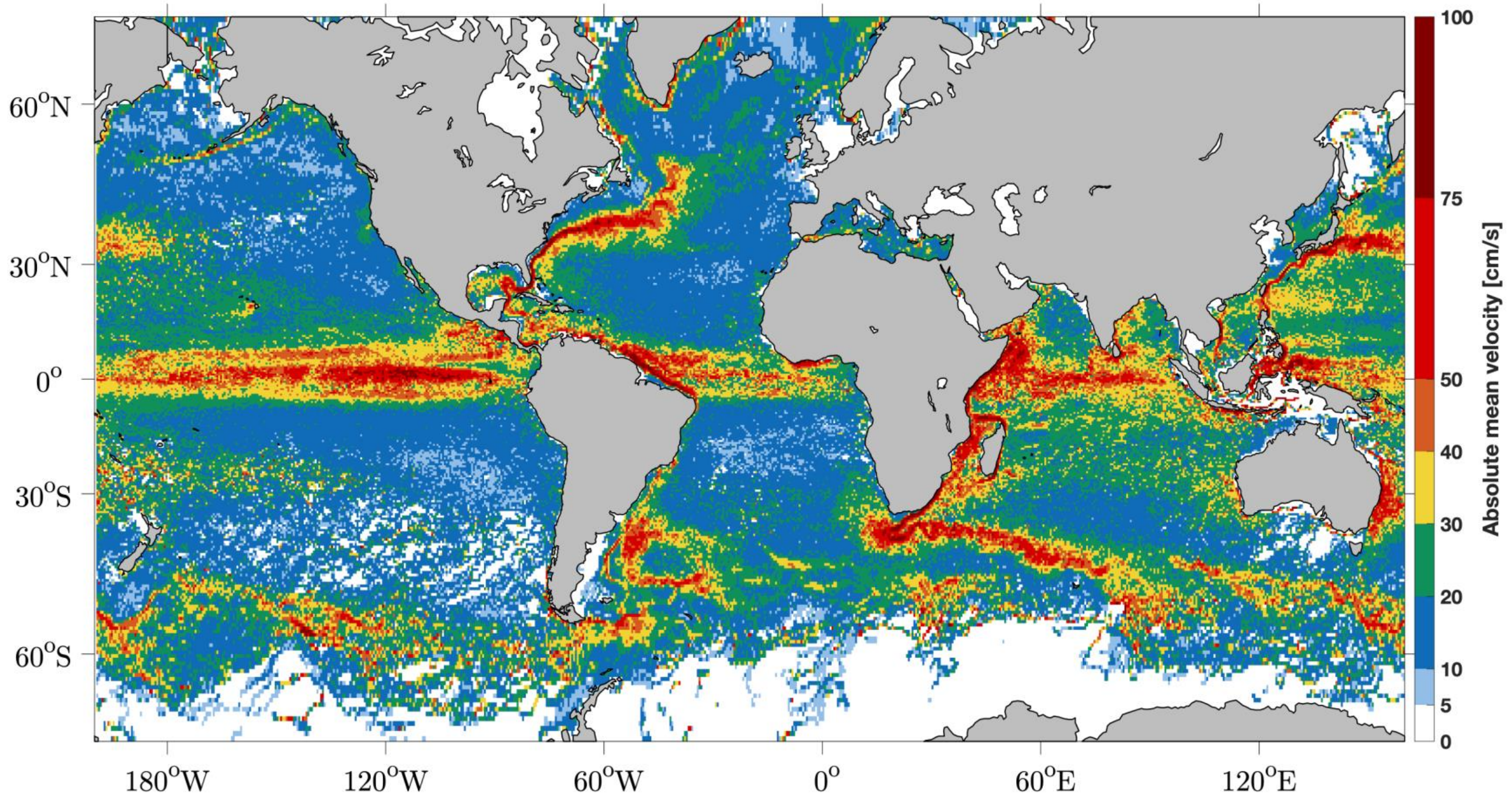

Supplementary Fig. 15 Global analysis of absolute mean velocity in cm/s derived from the Global Drifter Program dataset including 24,971 Surface Velocity Program (SVP) drifters. Drifter data were taken from reference 3.

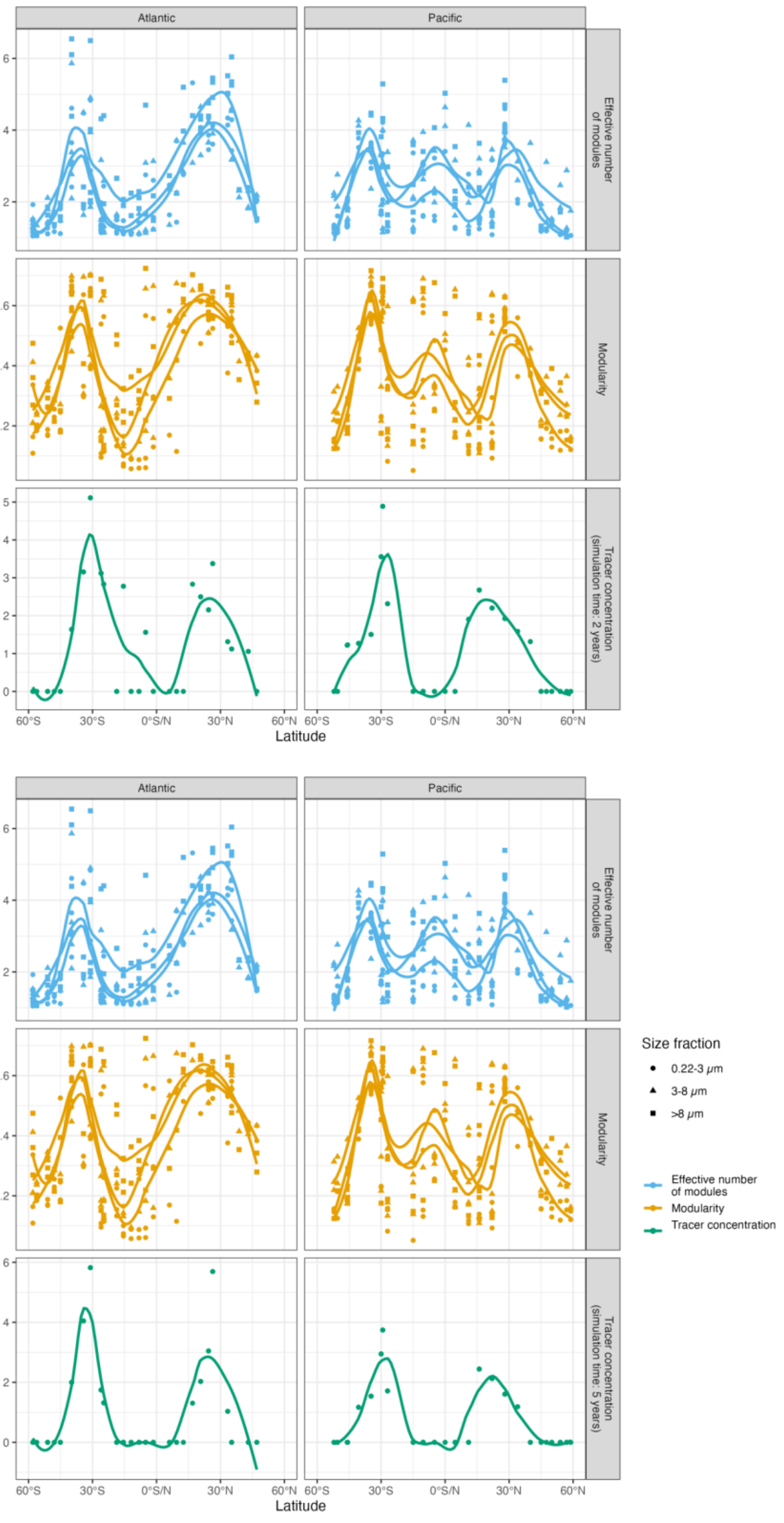

Supplementary Fig. 16 Overlap of tracer accumulation, sample modularity and effective module diversity along the Atlantic and Pacific Ocean transect for the upper epipelagic depth layer (20 m – DCM) and two different simulation times. Effective number of modules was calculated as the effective number of species (inverse Simpson) of the module abundance data. Modularity is derived from subnetworks of the global co-occurrence network based only on ASVs present in a single sample. Tracer concentrations were derived from the simulation of drifter advection. For that, rectangles were drawn around each sampling location and average tracer concentrations were retrieved for each rectangle. This analysis was done for 2 and 5 years simulation times to show how derived accumulation areas are affected. Source data are provided as a Source Data file.

References:

1. Milke, F. *et al.* Composition and biogeography of pro- and eukaryotic communities in the Atlantic Ocean: primer choice matters. *Front. Microbiol.* **13**, 895875 (2022).
2. Milke, F., Wagner-Doebler, I., Wienhausen, G. & Simon, M. Selection, drift and community interactions shape microbial biogeographic patterns in the Pacific Ocean. *ISME J.* **16,** 2653-2665 (2022) doi:10.1038/s41396-022-01318-4.
3. Elipot, S, Sykulski, A., Lumpkin, R, Centurioni, L., Pazos, M. Hourly location, current velocity, and temperature collected from Global Drifter Program drifters world-wide. NOAA National Oceanic and Atmospheric Administration. https://doi.org/10.25921/x46c-3620 (2019).
