# Supplementary material for: Ecological mechanisms and current systems shape the modular structure of the global oceans’ prokaryotic seascape
Source: Nat Commun. 2023 Oct 2;14:6141. doi: 10.1038/s41467-023-41909-z (PMC10545751; doi:10.1038/s41467-023-41909-z)
Supplement: Supplementary file 3 — Description of Additional Supplementary Files [file 41467_2023_41909_MOESM3_ESM.pdf]

Description of Additional Supplementary Files

Supplementary Data 1: List of ASVs of modules 1 to 10
