# Supplementary material for: Ecological mechanisms and current systems shape the modular structure of the global oceans’ prokaryotic seascape
Source: Nat Commun. 2023 Oct 2;14:6141. doi: 10.1038/s41467-023-41909-z (PMC10545751; doi:10.1038/s41467-023-41909-z)
Supplement: Supplementary file 5 — Reporting Summary [file 41467_2023_41909_MOESM5_ESM.pdf]

nature portfolio

Corresponding author(s): Meinhard Simon, Felix Milke

Last updated by author(s): August 30, 2023

Reporting Summary

Nature Portfolio wishes to improve the reproducibility of the work that we publish. This form provides structure for consistency and transparency in reporting. For further information on Nature Portfolio policies, see our Editorial Policies and the Editorial Policy Checklist.

Statistics

For all statistical analyses, confirm that the following items are present in the figure legend, table legend, main text, or Methods section.

n/a	Confirmed	
☐	☒	The exact sample size (*n*) for each experimental group/condition, given as a discrete number and unit of measurement
☐	☒	A statement on whether measurements were taken from distinct samples or whether the same sample was measured repeatedly
☐	☒	The statistical test(s) used AND whether they are one- or two-sided<br>*Only common tests should be described solely by name; describe more complex techniques in the Methods section.*
☐	☒	A description of all covariates tested
☐	☒	A description of any assumptions or corrections, such as tests of normality and adjustment for multiple comparisons
☐	☒	A full description of the statistical parameters including central tendency (e.g. means) or other basic estimates (e.g. regression coefficient) AND variation (e.g. standard deviation) or associated estimates of uncertainty (e.g. confidence intervals)
☐	☒	For null hypothesis testing, the test statistic (e.g. *F*, *t*, *r*) with confidence intervals, effect sizes, degrees of freedom and *P* value noted<br>*Give P values as exact values whenever suitable.*
☒	☐	For Bayesian analysis, information on the choice of priors and Markov chain Monte Carlo settings
☒	☐	For hierarchical and complex designs, identification of the appropriate level for tests and full reporting of outcomes
☐	☒	Estimates of effect sizes (e.g. Cohen's *d*, Pearson's *r*), indicating how they were calculated

Our web collection on statistics for biologists contains articles on many of the points above.

Software and code

Policy information about availability of computer code

Data collection	We used Qiime2 (version 2019.7), cutadapt (version 3.3), bbtools (version 38.7) and the SILVA database (version 132) to process 16S rRNA gene reads. Alignment was done with SINA (version 1.2.9) and trees were built with FastTree (version 2.1.11). Analyses were conducted in R (ASV tables with version 3.6.3, statistics with version 4.1.2) with packages vegan (version 2.6-4), igraph (version 1.5.1) and oceanmap (version 0.1.3). Drifter-Analyses were conducted with Matlab (version 2021a)

Data analysis	Nucleotide data was processed in Qiime2 using a set of published scripts (https://github.com/jcmcnch/eASV-pipeline-for-515Y-926R/releases/tag/v1.0.0). Shortly, 16S rRNA gene sequences were sequenced on an Illumina MiSeq (PE300) and denoised using DADA2 to infer Amplicon Sequence Variants (ASVs). ASVs were exported in a count table and further processed in R to calculate the following:<br>- alpha-diversity measures with vegan (richness and Effective Number of Species (inv. Simpson))<br>- compositional and phylogenetic beta-diversity matrices and their bootstrapping for inference of ecological mechanisms<br>- SparCC correlation measures between ASVs and their visualization in a network (using package igraph)<br>- clustering of network into modules<br>- subset network and calculation of subnetwork indices<br>- visualization of abundances of network modules<br>- constrained correspondance analysis (CCA)<br>- calculation of linear models<br>All R-scripts are published via Zenodo and Github (https://doi.org/10.5281/zenodo.8273515).<br>Drifter data were used to infer transport matrix after Mheen et al. (2019) using their published scripts (https://www.github.com/mheen/gdp_tm).

For manuscripts utilizing custom algorithms or software that are central to the research but not yet described in published literature, software must be made available to editors and reviewers. We strongly encourage code deposition in a community repository (e.g. GitHub). See the Nature Portfolio guidelines for submitting code & software for further information.

Data

Policy information about availability of data

All manuscripts must include a data availability statement. This statement should provide the following information, where applicable:

- Accession codes, unique identifiers, or web links for publicly available datasets
- A description of any restrictions on data availability
- For clinical datasets or third party data, please ensure that the statement adheres to our policy

Nucleotide data are deposited at the European Nucleotide Archive (ENA) under the following accession numbers:
- PRJEB51015 (Pacific Ocean transect) (https://www.ebi.ac.uk/ena/browser/view/PRJEB51015)
- PRJEB50983 (Atlantic Ocean transect) (https://www.ebi.ac.uk/ena/browser/view/PRJEB50983)
- PRJEB48162 & PRJEB35673 (SPOT time series) (https://www.ebi.ac.uk/ena/browser/view/PRJEB48162)
- PRJEB25224 (Malaspina data) (https://www.ebi.ac.uk/ena/browser/view/PRJEB35673)
- PRJEB44474 (Mediterranean Sea transect) (https://www.ebi.ac.uk/ena/browser/view/PRJEB44474)
Environmental data of the Pacific and Atlantic Ocean transect are deposited at PANGEA:
- Pacific Ocean: https://doi.pangaea.de/10.1594/PANGAEA.918500
- Atlantic Ocean: https://doi.pangaea.de/10.1594/PANGAEA.906247
Drifter data are available at the National Oceanic and Atmospheric Administration (NOAA) database: https://doi.org/10.25921/x46c-3620

Research involving human participants, their data, or biological material

Policy information about studies with human participants or human data. See also policy information about sex, gender (identity/presentation), and sexual orientation and race, ethnicity and racism.

Reporting on sex and gender	N/A
Reporting on race, ethnicity, or other socially relevant groupings	N/A
Population characteristics	N/A
Recruitment	N/A
Ethics oversight	N/A

Note that full information on the approval of the study protocol must also be provided in the manuscript.

Field-specific reporting

Please select the one below that is the best fit for your research. If you are not sure, read the appropriate sections before making your selection.

☐ Life sciences ☐ Behavioural & social sciences ☒ Ecological, evolutionary & environmental sciences

For a reference copy of the document with all sections, see nature.com/documents/nr-reporting-summary-flat.pdf

Ecological, evolutionary & environmental sciences study design

All studies must disclose on these points even when the disclosure is negative.

Study description	Analysis of 16S rRNA gene amplicon sequence variants (ASV, V4-V5 region) of samples collected at 22 stations in the Atlantic and Southern Ocean between 62°S and 47°N and of 26 station in the Pacific Ocean between 52°S and 59°N at depths between 20 and 200 m, covering all relevant biogeographic provinces. In addition 120 samples of the Malaspina expedition collected at 3 m depth in the Atlantic, Pacific and Indian Ocean, samples of a transect between the eastern Mediterranea Sea and the northwest Atlantic collected at 29 stations between 3 and 1000 m depth and at one station in the Pacific Ocean near the Californian coast (SPOT) collected monthly from 2005 to 2018 between 3 and 150 m were included in the analyses. Only environmental samples were collected without any further treatment factor, etc. 20 L-water samples were collected at each depth without any replicates.
Research sample	Water samples containing microorganisms which were size-fractionated into 0.2-3.0 µm (free-living prokaryotes), 3-8 and >8 µm (both particle-associated prokaryotes). These size fractions are only available for the samples of the Atlantic and Pacific Ocean. Samples from the SPOT station in the Pacific Ocean were size-fractionated into 0.2-1.0 and >1 µm. Samples from the Malaspina expedition and the Mediterranean Sea were size-fractioned to 0.22-3 µm. For data sources see study description and Data statement. In each sample, the prokaryotic microbial community was analysed based on the V4-V5 region of the 16S rRNA gene. We were aiming to sample open ocean prokaryotic communities with a broad spatial coverage, including not only the Atlantic and Pacific Ocean basins, but also the other parts of the global oceans by adding data from other expeditions.
Sampling strategy	We aimed to cover a representative set of biogeographic provinces in the Atlantic and Pacific Ocean and to include major regions of other oceans and the Mediterranean Sea. Regarding our own sampling strategy we were constraint in the number of stations per biogeographic region by ship time. Samples were taken from transects that aimed to cover a broad latitudinal range from southern to northern subpolar regions. Stations were distributed evenly along these latitudinal transects.
Data collection	We collected water samples using a CTD rosette with mounted 20 L Niskin bottles on the RVs Sonne and Polarstern. 10 L of seawater from depths of 20, 40, 60, 100 and 200 m and the deep chlorophyll maximum (DCM) were sequentially filtered through 8, 3 and 0.22µm filters to retrieve the three size-fractionated microbial communities on the filters. Filters were frozen at -80°C until further processing in the laboratory in Oldenburg, Germany. There, filters were thawed and DNA was extracted from a subset of these filters. Extracts were amplified using V4-V5 16S rRNA gene primers and sequenced on an Illumina MiSeq (PE300).
Timing and spatial scale	Atlantic/Southern Ocean: Sampling at the stations in the Southern Ocean started on March 16, 2012 and ended at the northernmost station in the Atlantic on May 11, 2012.<br>Pacific Ocean: Sampling was done between May 3rd and 31st 2016 (30°S to 59°N) and between 1 and 15 February 2017 (30°S and 52°S).<br>For sampling times of the Malaspina expedition, the Mediterranean Sea and at SPOT see respective publications.
Data exclusions	No data were excluded.
Reproducibility	The analysis of samples from a single station and depth are based on a single sample. Due to logistic and financial constraints replicate samples could not be taken. This procedure is quite normal in these types of analyses.
Randomization	Not applicable because the ocean regions were sampled systematically along the transects and downloaded samples from other ocean regions needed to cover systematically these regions
Blinding	Not applicable. Blinding is never done in such biogeographic analyses, as spatial and temporal data must be analyzed systematically and context sensitive.

Did the study involve field work? ☒ Yes ☐ No

Field work, collection and transport

Field conditions	Oceanographic study, in the Atlantic Ocean between subantarctic and boreal regions (52°S and 47°N) and in the Pacific Ocean between subantarctic and subarctic regions (52°S and 59°N) based an samples collected by a rosette sampler from RV Polarstern and RV Sonne, rough to smooth sea conditions from sea surface temperatures around 0°C to 30°C.
Location	Sampling stations in the Atlantic Ocean were conducted from 62°S to 47°N, reaching from the Southern Ocean towards the South Atlantic Gyre, the equatorial region and into the North Atlantic Gyre. The sampling stations in the Pacific Ocean were taken from 52°S to 59°N mostly along 180°E/W with few exceptions (around New Zealand). The exact transects and sampling locations are marked in figure 1a in the publication At each stations we sampled seawater at depths of 20, 40, 60, 100, DCM and 200m. If DCM was overlapping with one of the other regular sampled depths they were combined.
Access & import/export	Most work was in international waters, several stations in the tropical and subarctic Pacific and Bering Sea in the EEZ of France and the USA, several stations were in the EEZ of New Zealand. Permission was approved. Wiss 462.26 (FR), LGL/MSR/2015-009 (NZ), U2016-005 (US). We only dealt with frozen and no live samples.
Disturbance	Collection of water in rather distant oceanic regions does not cause any disturbance (except chasing a few pelagic birds).

Reporting for specific materials, systems and methods

We require information from authors about some types of materials, experimental systems and methods used in many studies. Here, indicate whether each material, system or method listed is relevant to your study. If you are not sure if a list item applies to your research, read the appropriate section before selecting a response.

Materials & experimental systems

n/a	Involved in the study
☒	☐ Antibodies
☒	☐ Eukaryotic cell lines
☒	☐ Palaeontology and archaeology
☒	☐ Animals and other organisms
☒	☐ Clinical data
☒	☐ Dual use research of concern
☒	☐ Plants

Methods

n/a	Involved in the study
☒	☐ ChIP-seq
☒	☐ Flow cytometry
☒	☐ MRI-based neuroimaging
